# Function and regulation of miR-186-5p, miR-125b-5p and miR-1260a in chordoma

**DOI:** 10.1186/s12885-023-11238-x

**Published:** 2023-11-27

**Authors:** Xulei Huo, Ke Wang, Bohan Yao, Lairong Song, Zirun Li, Wenyan He, Yiming Li, Junpeng Ma, Liang Wang, Zhen Wu

**Affiliations:** 1 Department of Neurosurgery, Beijing Tiantan Hospital, Capital Medical University, Nansihuanxilu 119, Fengtai District, Beijing, 100070 China; 2 China National Clinical Research Center for Neurological Diseases, Beijing, China; 3 Department of Neurosurgery, Tianjin Medical University General Hospital, TianJin, China

**Keywords:** Chordoma, miRNA, Migration, Proliferation, mRNA

## Abstract

**Background:**

The function and regulation of miRNAs in progression of chordoma were unclear.

**Methods:**

Five miRNAs were identified by the machine learning method from the miRNA expression array. CCk-8 assay, EDU assay, wound healing migration assay, and trans-well assay were used to reveal the effect of the miRNAs in chordoma cell lines. Moreover, bioinformation analysis and the mRNA expression array between the primary chordomas and recurrent chordomas were used to find the target protein genes of miRNAs. Furthermore, qRT-PCR and luciferase reporter assay were used to verify the result.

**Results:**

miR-186-5p, miR-30c-5p, miR-151b, and miR-125b-5p could inhibit proliferation, migration, and invasion of chordoma while miR-1260a enhances proliferation, migration, and invasion of chordoma. Recurrent chordoma has a worse disease-free outcome than the primary chordoma patients. AMOT, NPTX1, RYR3, and P2RX5 were the target protein mRNAs of miR-186-5p; NPTX1 was the target protein mRNAs of miR-125b-5p; and AMOT and TNFSF14 were the target protein mRNAs of miR-1260a.

**Conclusions:**

miR-186-5p, miR-125b-5p, miR-1260a, and their target protein mRNAs including AMOT, NPTX1, RYR3, P2RX5, TNFSF14 may be the basement of chordoma research.

**Supplementary Information:**

The online version contains supplementary material available at 10.1186/s12885-023-11238-x.

## Introduction

Chordoma is a malignant bone tumor, which develops from the remnants of notochord [1] and mainly occurs at skull-base and sacrococcygeal [2]. Generally, chordoma has a high recurrence rate and a low progression free survival rate, although it grows slowly, especially the skull-base cases[3]. In addition, surgery and chemotherapy are the main therapeutic strategies for chordoma patients. Drug resistance or the limited number of drugs remarkably limit the application of newly developed biological therapy and targeted therapy[4]. Therefore, more study on the malignant biological behaviour and the establishment of progression blockage are necessary and urgent for the treatment of chordoma.

Until now, miRNAs have been shown a crucial role in the development of central neuro system neoplasms, including glioma, meningioma, chordoma[5–8]. Previous study has proven that miR-574-3p, miR-1237-3p, miR-140-3p, miR-1, miR-155, and miR-1290 could be prognostic factors for the spinal chordomas[7]. Moreover, Met is a proto-oncogene that overexpressing in chordoma, and miR-1 could suppress migratory and invasive activities of chordoma cells by downregulating Met[9]. Furthermore, miR-1 was also associated with the clinical outcome of chordoma[10]. In addition, overexpression of miR-16-5p could inhibit proliferation, invasion and migration of chordoma and was associated with the upregulated expression of E-cadherin and downregulated expression of N-cadherin and vimentin [8]. In another study, the authors revealed that LOC554202 may play an important role in the progression of chordoma by the direct upregulation of EZH2 and indirect promotion of RNF144B via miR-31[11]. However, the detailed molecular mechanisms of miRNAs in chordoma have not been elucidated clearly.

In this study, we developed a machine learning method to identified the underlying subgroups in chordoma to determine important miRNAs in development and progression of chordoma. After the functional experiment of these miRNAs, we found miR-186-5p, miR-30c-5p, miR-151b, and miR-125b-5p could inhibit proliferation, migration, and invasion of chordoma while miR-1260a enhances proliferation, migration, and invasion of chordoma. Furthermore, AMOT, NPTX1, RYR3, and P2RX5 were identified the target protein mRNAs of miR-186-5p; NPTX1 was identified the target protein mRNAs of miR-125b-5p; and AMOT and TNFSF14 were identified the target protein mRNAs of miR-1260a. These finding revealed the role and regulation mechanisms of five miRNA including four miRNA acting as anticancer role and one miRNA acting as cancer role.

## Materials and methods

### Patient cohort

Fifity chordoma patients included in this study were enrolled, who underwent resection at Beijing Tiantan Hospital. Patients were operated on between 2010 and 2019. All patients included in this study were diagnosed with classic chordomas based on pathological examination, which was consistent with the classification of bone tumors according to the latest 2021 WHO guidelines. The chart review including age, gender, adjuvant radiotherapy, extent of surgery, and disease-specific survival (DFS) was used to assemble clinical history of the patients retrospectively. The extent of resection was identified based on surgical record information and the postoperative magnetic resonance imaging (MRI) within one month. The definition of gross total resection (GTR) was no evidence of residual neoplasm intraoperatively and postoperative MRI. Conversely, definition of subtotal resection was any evidence of residual neoplasm intraoperatively or postoperative MRI. DFS was set the internal between the surgery time and the event that death or progression. And, we selected twelve samples randomly from the fifty samples for the Whole-Transcriptome Sequencing.

All tissues were gathered immediately after the surgery, snap frozen in liquid nitrogen. Reviewers that blinded to the original diagnosis did histological diagnoses on formalin - fixed, paraffin-embedded hematoxylin and eosin sections.

### Whole-transcriptome construction and sequencing

Total RNA was extracted with the Trizol reagent kit (Invitrogen, Carlsbad, CA, USA) according to the instruction. Agilent 2100 Bioanalyzer (Agilent Technologies, Palo Alto, CA, USA) and RNase free agarose gel electrophoresis were used to assess RNA quality. rRNAs were eliminated from the total RNA. The enriched mRNAs and ncRNAs were cut into fragments and converse transcribed into cDNA. Then, after the synthesize of second-strand cDNA, QiaQuick PCR extraction kit (Qiagen, Venlo, The Netherlands) was used to purifie the cDNA items. And, the items were added poly(A) and ligated to Illumina adapters. Lastly, the second strand cDNA chain was removed by Uracil-N-Glycosylase. The digested items were screened, PCR magnified, and sequenced with the Illumina HiSeq TM 4000 (Gene Denovo Biotechnology Co., Guangzhou, China).

Library preparation for small RNA sequencing: total RNA was extracted, the RNA size range of 18–30nt were enriched by polyacrylamide gel electrophoresis. Then, 36-44nt RNAs were enriched after 3’ adapters added. The ligation products that RNAs ligating the 5’ adapters were converse transcribed by PCR amplification. After that,140-160 bp size PCR items were enriched to produce a cDNA library. Illumina HiSeq TM 2500 in Gene Denovo Biotechnology Co. (Guangzhou, China) was utilized to sequence.

### Identification of differentially expressed miRNAs

Raw reads were removed the adapter by cutadapt (version 3.4) to acquire clean reads. miRNA was mapped to reference genome Human hg38 by mirdeep2.pl. Deseq2 R package (version 1.34.0) was applied to identify the significant differential RNA[12]. K-means consensus clustering was performed and visualized in a heatmap with associated dendrograms. Then, different miRNAs were identified with the threshold of Benjamini and Hochberg P values 0.05 and | log change | ≥ 1.

### Cell culture and transfection

The human chordoma cell lines, U-CH1 and UM-chor1, were used as the published study[13]. The culture medium was Iscove’s Modified Dulbecco’s Medium (Gibco [Life Technologies, Thermo Fisher Scientific]): Roswell Park Memorial Institute (Gibco [Life Technologies, Thermo Fisher Scientific]) 1640 (4:1) and 10% fetal bovine serum (Gibco [Life Technologies, Thermo Fisher Scientific]), 1% penicillin and streptomycin (100 mg/ mL; Invitrogen). Cells were maintained in a humidified incubator containing 5% CO_2_ and 95% air atmosphere at 37 °C. U-CH1 and UM-Chor1 cells were seeded in 6-well plate with a density of 15 × 10^4^ cells. After 24 h, cells were transfection with negative control (NC), miR-186-5p, miR-30c-5p, miR-151b, miR-125b-5p, miR-1260a mimics and miR-186-5p, miR-125b-5p, miR-1260a inhibitors (RiboBio, Guangzhou, China). The concentration was 50 nM.

### Quantitative real-time polymerase chain reaction

Total RNA was extracted with miRcute miR Isolation Kit (Tiangen, Beijing, China) or RNAsimple Total RNA Kit (Tiangen, Beijing, China) in accordance with manufacturer’s protocol from the 6-well plates. Primers of mRNAs and miRNAs were purchased from Biomed (Beijing, China) or Tiangen company. RNA was quantified by NanoDrop ND-1000 spectrophotometer (Thermo-Scientific, Waltham, MA, USA). For miRNA, Equal amounts of total RNA (800 ng) were reverse-transcribed using the miRcute Plus miR First-Strand cDNA Kit (Tiangen, Beijing, China) and the expression levels was quantified by miRcute Plus miR qPCR Kit (Tiangen, Beijing, China). For mRNA, Equal amounts of total RNA (500 ng) were reverse-transcribed using the PrimeScript™ RT Master Mix (Takara, Beijing, China) and the expression levels was quantified by using TB Green® Premix Ex Taq™ II (Takara, Beijing, China). In addition, the experiment was performed as described [14]. Relative expression levels of miRNA and mRNA were normalized to the mean of the NC groups. The 2^−ΔΔCt^ method was used for data analysis. U6 and GAPDH was used as reference genes.

### Cell proliferation assay

Transfected U-CH1 and UM-Chor1 cells were seeded in 96-well plates with 3 × 10^3^ cells per well in duplicates. Cell viability was assessed at 4, 24, 48, 72, 96, and 120 h with Cell Counting Kit-8 (CCK-8, Dojindo Molecular Technologies, Inc., Kumamoto, Japan). Absorbance was measured at 450 nm with the Tecan Spark Microplate Reader (Tecan Group AG, Mannedorf, Switzerland).

In addition, 5-ethynyl-20-deoxyuridine (EdU) assay kit (Solarbio, Beijing, China) was applied to inquire the cell proliferation ability. Cells were seeded into 96-well plates with a density of 4 × 10^4^ cells. After 24 h, the cell monolayer was washed carefully with 100 µL PBS to remove cell debris and thereafter incubated with 50 µM EdU buffer at 37 °C for 2 h, fixed with 4% formaldehyde for 40 min and stained nuclei with Hoechst in accordance with the manufacturer’s protocol. Then the results were visualized by a fluorescence microscope.

### Wound healing assay

The migratory characteristics of U-CH1 and UM-Chor1 were determined by scratch wound assay. Transfected cells and their negative control were seeded in a 24-well culture plate (10 × 10^4^ cells) and cultured to a confluent state. Artificial wound was introduced with a P-200 pipette tip in each well and rinsed with PBS to remove detached cells. U-CH1 and UM-Chor1 were allowed to migrate for 24 and 48 h. Photographs were subsequently taken through an inverted microscope (10 X).

### Transwell invasion assay

Migration or invasion assays were performed with 8-µm pore size transwell filter insert (Costar 3422 [Corning, Corning, New York, USA]) with or without pre-coated diluted Matrigel (1:8) (Becton Dickinson and Co., Franklin Lakes, NJ, USA). 5 × 10^4^ transfected cells with Serum-free medium were placed into the upper chamber, and 500 µL medium containing 20% FBS was added into the bottom chamber. After incubation in 37 °C for 24 h, cells on the underside of membrane were immobilized about 40 min with 4% Paraformaldehyde (Solarbio, Beijing, China) and stained about 30 min with 0.4% crystal violet (Solarbio, Beijing, China). The penetrated cells were counted in five random fields. NC group was used as reference group.

### Prediction of mRNAs of miRNA

The mRNA and miRNA pairs were generated by multiMiR R package [15] with criterion that the pair was validated and the other parameters were default. For improving prediction accuracy of the five miRNAs, a mRNA array contrast was performed between 25 primary chordoma patients and 25 recurrent chordoma patients. The adapter of raw reads was removed by fastp [16] (version 0.20.1) to obtain clean reads. The rRNA was removed by Bowtie2 (version 2.4.4) [17]. The mRNA fastq sequences were mapped to reference genome Human GRCh38 by hisat2 [18] (version 2.2.1). Differentially mRNAs were identified with the threshold of | log change | ≥ 2 and Benjamini and Hochberg *p* value < 0.05. Venn diagram showed the overlap of predicting genes and differentially mRNAs. Also, RT-PCR experiments were used to confirm the results.

### Luciferase reporter assay

The AMOT/NPTX1 3′-UTR containing the wild-type or mutated miR-186-5p/miR-125b-5p binding sequences was synthesized by RiboBio (Guangzhou, China) and cloned into the pmiR-RB-REPORT vector (RiboBio). U-CH1 cells were transfected with the wild- type/mutant AMOT/NPTX1 luciferase reporter vector and miR-186-5p/miR-125b-5p mimic or negative control using Lipofectamine 3000. Luciferase activity was measured using a Dual-Luciferase Reporter Assay System (E2920, Promega), and the results are expressed as firefly luciferase activity normalized to Renilla luciferase activity.

### Statistical analysis

All statistical analyses were performed with R (version 4.1.2) or GraphPad Prism (version 9.0.0, GraphPad, San Diego, CA, USA). The results from Edu assay and transwell assay were analysed by ImageJ (version2.3.0, National Institutes of Health, Bethesda, MD, USA). Continuous variables were analyzed using independent sample t test or Mann-Whitney U test for the parametric and nonparametric variables, respectively. Chi- Square test was used to compare the differences in categorical variables. * P < 0.05; ** P < 0.01; *** P < 0.001.

## Results

### Identification of miRNA clusters

**Fig. 1:**
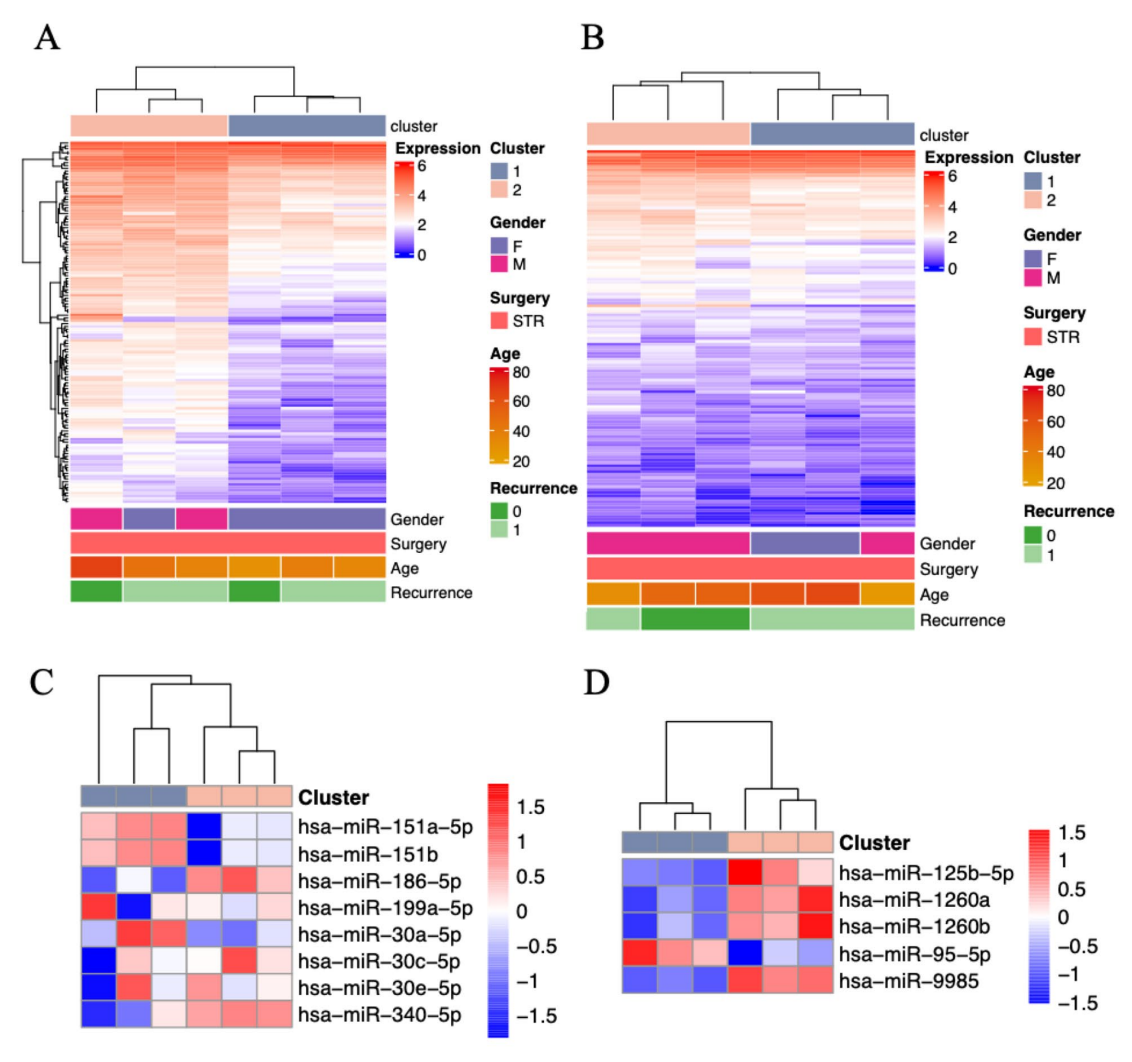
Identification of miRNA clusters. **A** and **B**. Identification of miRNA clusters. **C** and **D**. miR-30e-5p, miR-340-5p, miR-186-5p, miR-30c-5p, miR-199a-5p, miR-30a-5p, miR-151a-5p, and miR-151b were revealed while in the Fig. 1B, miR-95-5p, miR-1260a, miR-1260b, miR-125b-5p, and miR-9985 were revealed

The expression levels of RNAs in the twelve chordoma samples were explored. And, the twelve samples were divided into two groups with their corresponding clinical information were showed in the Fig. 1. After selecting the pre-treated data by adjusted p values 0.05 and | log change | ≥ 1, we got 13 different expressed miRNAs (Fig. 1A and B). In the Fig. 1A, miR-30e-5p, miR-340-5p, miR-186-5p, miR-30c-5p, miR-199a-5p, miR-30a-5p, miR-151a-5p, and miR-151b were revealed while in the Fig. 1B, miR-95-5p, miR-1260a, miR-1260b, miR-125b-5p, and miR-9985 were revealed. The 13 miRNAs were validated in the following procedures. And, we chosen miR-186-5p, miR-30c-5p, miR-151b, miR-125b-5p, and miR-1260a as our study targets.

### Mir-186-5p inhibits proliferation, migration, and invasion of chordoma

**Fig. 2:**
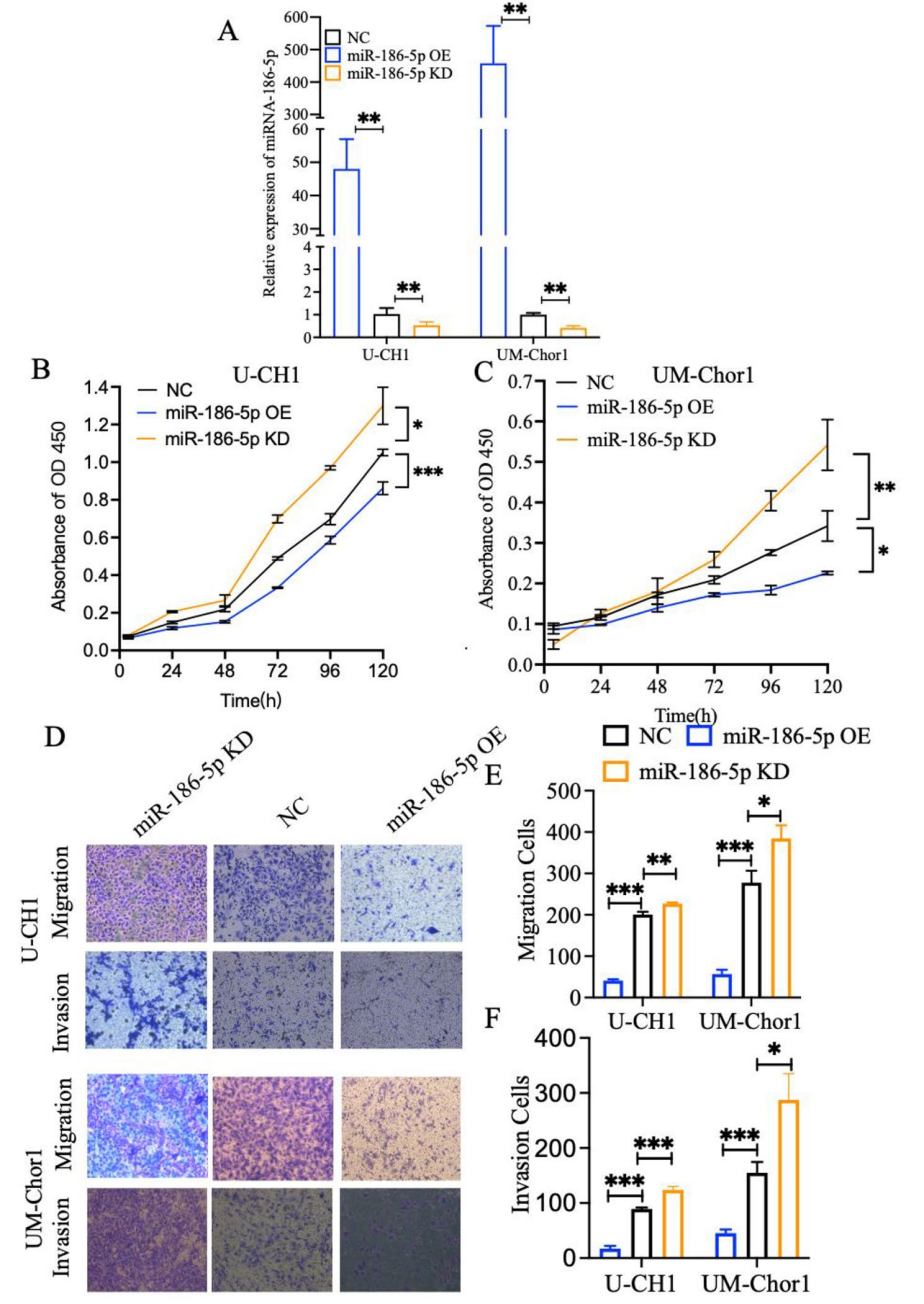
miR-186-5p inhibits the migration, invasion and proliferation of U-CH1 cells and UM-Chor1 cells. **A** qRT - PCR results of miR-186-5p in U-CH1 cells and UM-Chor1 cells from the miR-186-5p overexpression (miR-186-5p OE) group, miR-186-5p knockdown (miR-186-5p KD) group and NC group. U6 was served as the internal control. **B** Representative pictures of real-time proliferation assay in U-CH1 cells and **C** UM-Chor1 cells from the miR-186-5p OE group,miR-186-5p KD, and the NC group. **D** Representative pictures and **E, F** quantitative data of transwell assay in U-CH1 cells and UM-Chor1 cells from the miR-186-5p OE group, miR-186-5p KD, and NC group. Each experiment was performed in in triplicate and repeated three times. Results are presented as mean ± standard deviation

For further identify the effect of miR-186-5p on chordoma cells, chordoma cells were transfected with miR-186-5p mimics and the miR-186-5p inhibitors. qRT-PCR result identified that the miR-186-5p overexpression (OE) group has significantly high expression level than NC group and miR-186-5p knockdown (KD) group has significantly low expression level than NC group in U-CH1 and UM-chor1 cells (Fig. 2A). Then, CCK-8 assay showed that the proliferation of U-CH1 and UM-chor1 cells was significantly inhibited and enhanced in the miR-186-5p OE group and miR-186-5p KD group compared with NC group (Fig. 2B, and 2 C). And, EDU assay showed that the proliferation of U-CH1 and UM-chor1 cells was significantly inhibited in the miR-186-5p OE group compared with NC group (Figure S1A, and S1B). In addition, transwell experiment indicated that the migration and invasion ability of chordoma could attenuated by miR-186-5p (Fig. 2D and E F, S1C, S1D, S1E and S1F). These results collectively suggested that miR-186-5p inhibits cell proliferation, migration, and invasion.

### miR-125b-5p inhibits proliferation, and invasion of chordoma

**Fig. 3:**
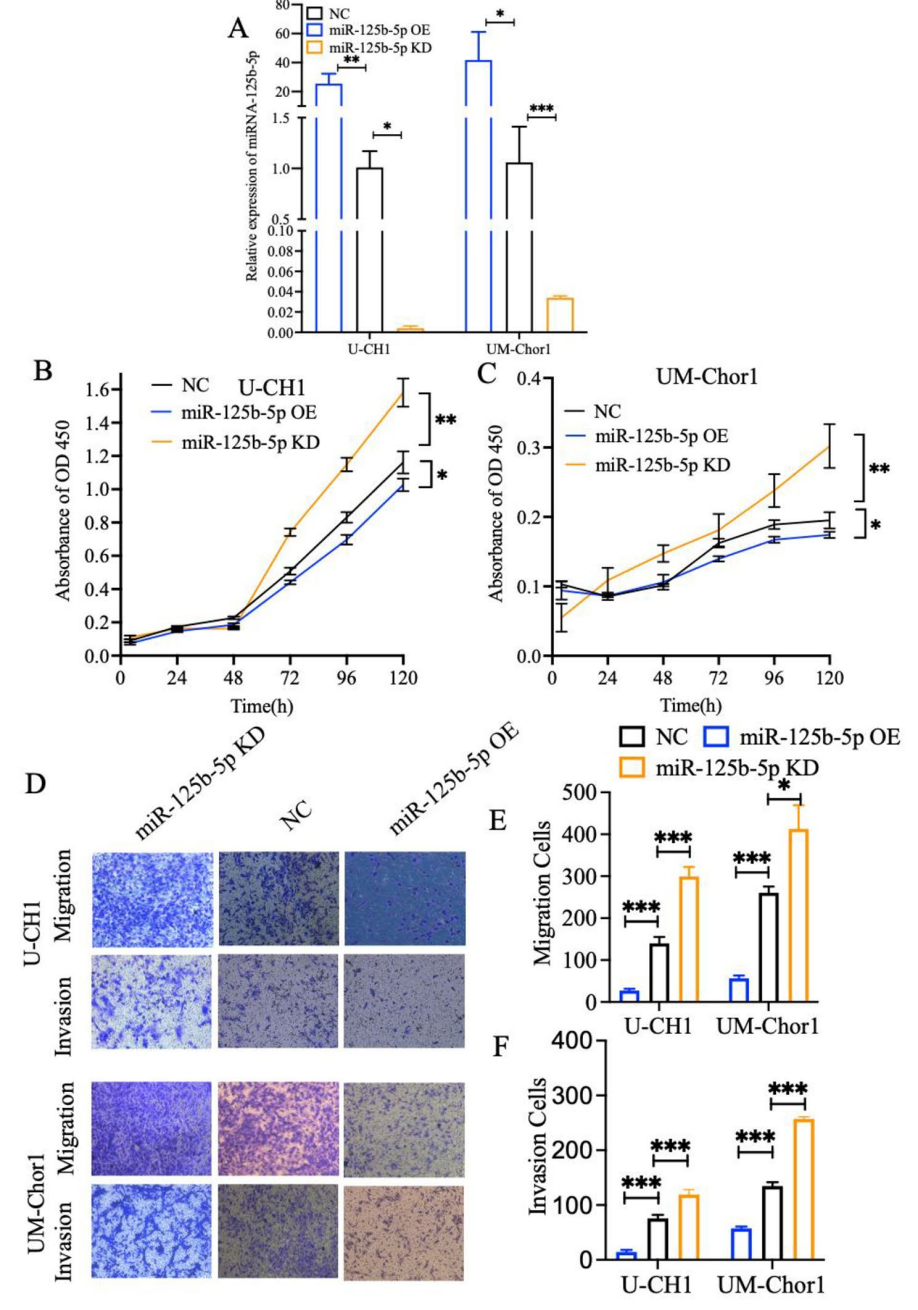
miR-125b-5p inhibits the migration, invasion and proliferation of U-CH1 cells and UM-Chor1 cells. **A** qRT - PCR results of miR-125b-5p in U-CH1 cells and UM-Chor1 cells from the miR-125b-5p overexpression (miR-125b-5p OE) group, miR-125b-5p knockdown (miR-125b-5p KD) group, and NC group. U6 was served as the internal control. **B** Representative pictures of real-time proliferation assay in U-CH1 cells and **C** UM-Chor1 cells from the miR-125b-5p OE group,miR-125b-5p KD group, and the NC group. **D** Representative pictures and **E, F** quantitative data of transwell assay in U-CH1 cells and UM-Chor1 cells from the miR-125b-5p OE group, miR-125b-5p KD group, and NC group. Each experiment was performed in in triplicate and repeated three times. Results are presented as mean ± standard deviation

For further identify the effect of miR-125b-5p on chordoma cells, chordoma cells were transfected with miR-125b-5p mimics and the miR-186-5p inhibitors. qRT-PCR result revealed that the miR-125b-5p OE group has significantly high expression level than NC group and miR-125b-5p KD group has significantly low expression level than NC group in U-CH1 and UM-chor1 cells (Fig. 3A). Then, CCK-8 assay showed that the proliferation of U-CH1 and UM-chor1 cells was significantly inhibited and enhanced in the miR-125b-5p OE group and miR-125b-5p KD group compared with NC group (Fig. 3B, and 3 C). And, EDU assay showed that the proliferation of U-CH1 and UM-chor1 cells was significantly inhibited in the miR-125b-5p OE group compared with NC group (Figure S2A, and S2B). In addition, wound healing assay and transwell experiment indicated that the migration and invasion ability of chordoma could attenuated by miR-125b-5p (Fig. 3D and E F, S2C, S2D, S2E and S2F). These results collectively suggested that miR-125b-5p inhibits cell proliferation, migration, and invasion.

### miR-1260a enhances proliferation, and invasion of chordoma

**Fig. 4:**
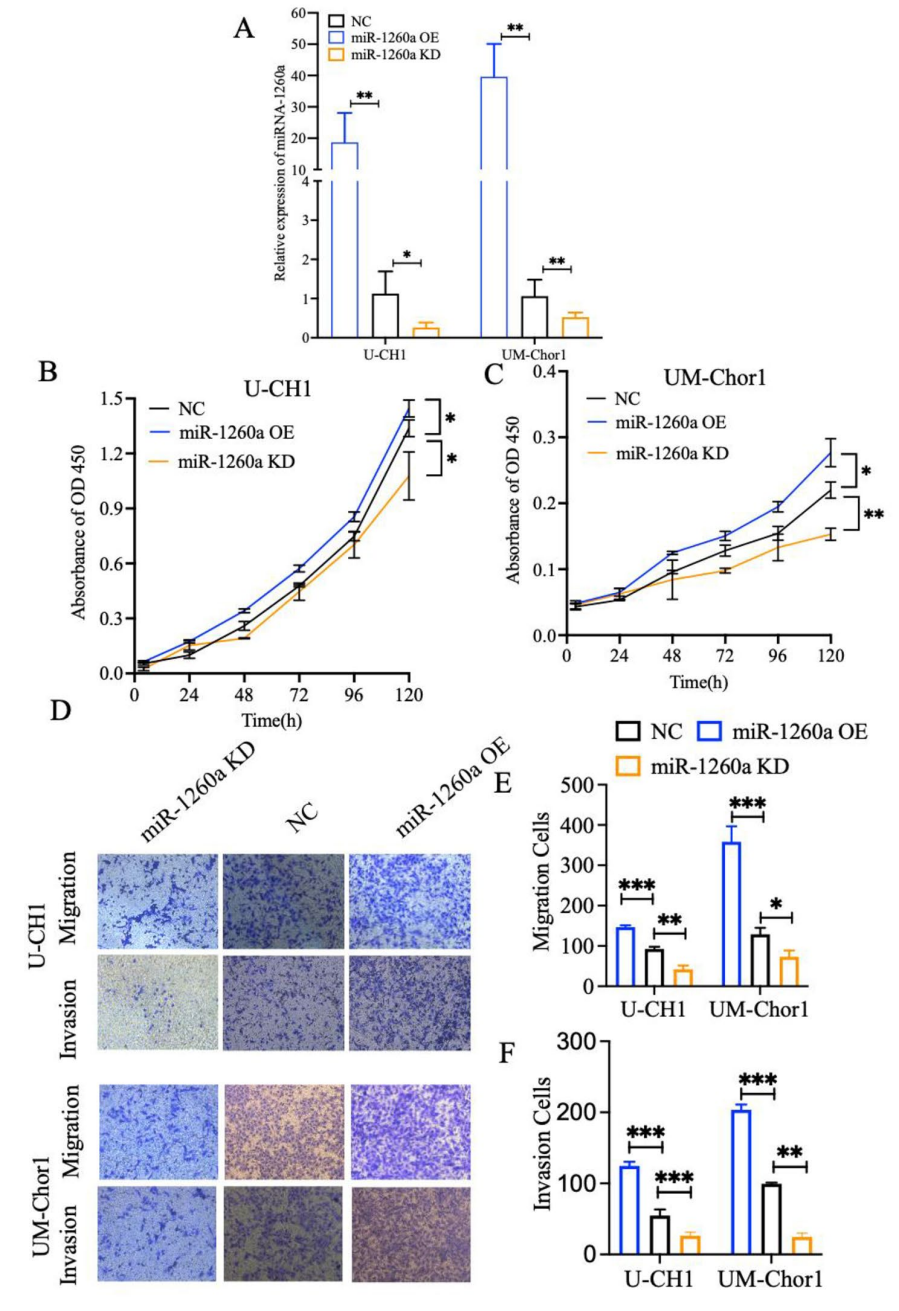
miR-1260a enhances the migration, invasion and proliferation of U-CH1 cells and UM-Chor1 cells. **A** qRT - PCR results of miR-1260a in U-CH1 cells and UM-Chor1 cells from the miR-1260a overexpression (miR-1260a OE) group, miR-1260a knockdown (miR-1260a KD) group, and NC group. U6 was served as the internal control. **B** Representative pictures of real-time proliferation assay in U-CH1 cells and **C** UM-Chor1 cells from the miR-1260a OE group, miR-1260a KD group, and the NC group. **D** Representative pictures and **E, F** quantitative data of transwell assay in U-CH1 cells and UM-Chor1 cells from the miR-1260a OE group, miR-1260a KD group, and NC group. Each experiment was performed in in triplicate and repeated three times. Results are presented as mean ± standard deviation

For further identify the effect of miR-1260a on chordoma cells, chordoma cells were transfected with miR-1260a mimics and the miR-186-5p inhibitors. qRT-PCR r result revealed that the miR-1260a OE group has significantly high expression level than NC group and miR-1260a KD group has significantly low expression level than NC group in U-CH1 and UM-chor1 cells (Fig. 4A). Then, CCK-8 assay showed that the proliferation of U-CH1 and UM-chor1 cells was significantly inhibited and enhanced in the miR-125b-5p KD group and miR-125b-5p OE group compared with NC group (Fig. 4B, and 4 C). And, EDU assay showed that the proliferation of U-CH1 and UM-chor1 cells was significantly elevated in the miR-125b-5p OE group compared with NC group (Figure S3A, and S3B). In addition, wound healing assay and transwell experiment indicated that migration and invasion ability of chordoma were elevation by miR-1260a transfected cells (Fig. 4D and E F, S3C, S3D, S3E and S3F). These results collectively suggested that miR-1260a could enhance chordoma cell proliferation, migration, and invasion.

### Identification of target protein mRNA of miRNA

**Fig. 5:**
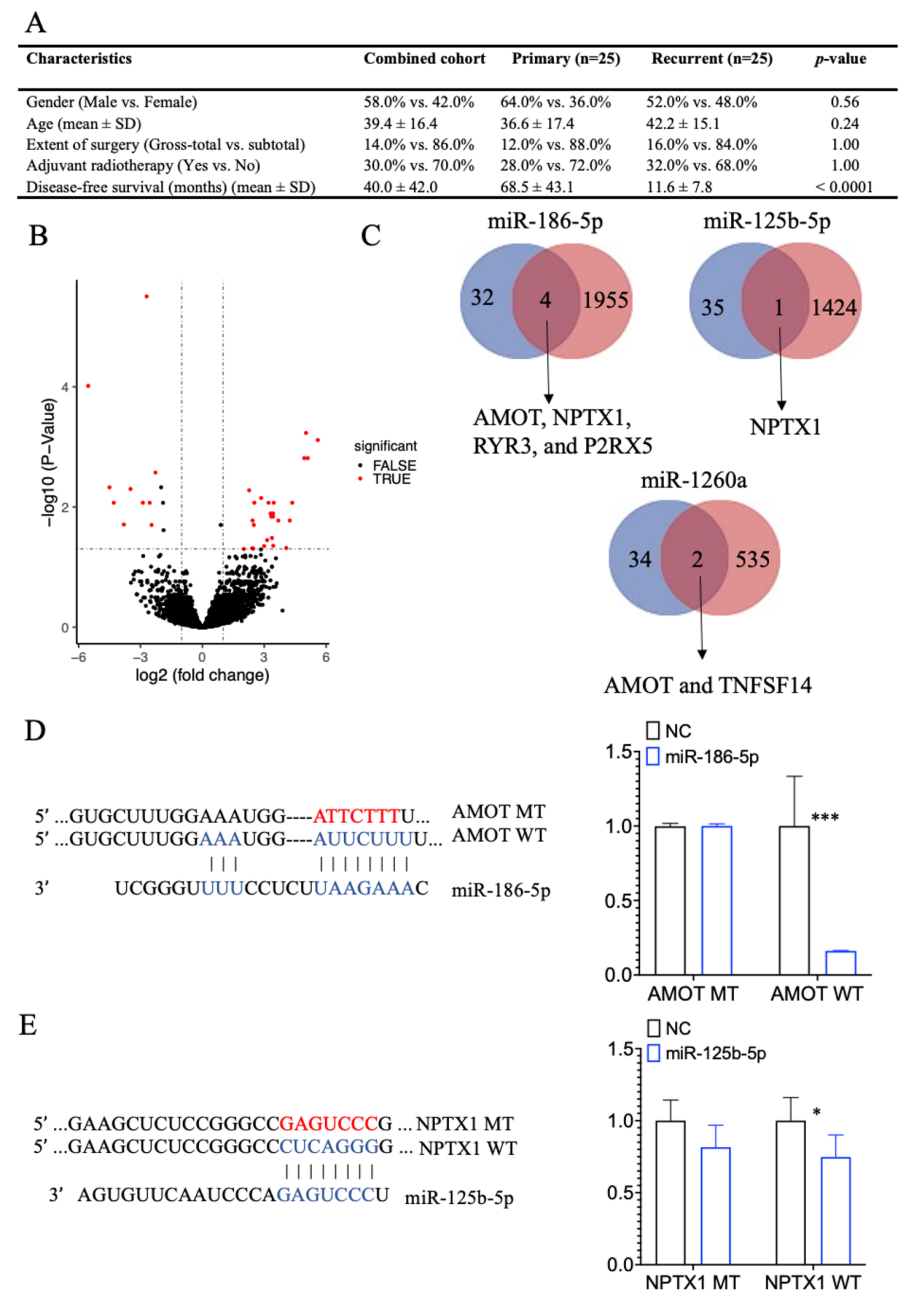
Target protein mRNAs of miR-186-5p, miR-125b-5p and miR-1260a. **A**. Clinical information of primary chordomas and recurrent chordomas. **B**. Volcano plot picturing different expressed mRNAs between primary chordomas and recurrent chordomas. Bioinformation analysis **C** revealed that AMOT, NPTX1, RYR3, and P2RX5 were the target protein mRNAs of miR-186-5p; NTPTX1 was the target protein mRNAs of miR-125b-5p; AMOT and TNFSF14 were the target protein mRNAs of miR-1260a. GADPH was served as the internal control. Each experiment was performed in in triplicate and repeated three times. Results are presented as mean ± standard deviation. **D** a Schematic representation of AMOT 3′-UTR containing the wild-type or mutant binding site for miR-186-5p and Luciferase reporter activity following expression after transfection (mimic control and miR-186-5p mimic) in U-CH1 cells. **E** a Schematic representation of ENPTX1 3′-UTR containing the wild-type or mutant binding site for miR-125b-5p and Luciferase reporter activity following expression after transfection (mimic control and miR-125b-5p mimic) in U-CH1 cells

Baseline clinical information of 50 chordoma samples including primary and recurrent chordomas were displayed in Fig. 5A. There is no difference in the gender, age, extent of surgery, and adjuvant radiotherapy between the two clusters. In addition, primary chordoma patients had a statistically significant better disease-free survival than recurrent chordoma patients (P < 0.0001). The mean survival time was 68.5 years in primary chordoma patients while recurrent chordoma patients was 11.6 years.

A volcano plot of probes displayed that the 36 differentially mRNAs were those had | log change | ≥ 2 and Benjamini and Hochberg P value < 0.05 (Fig. 5B) between.

8 primary chordoma and 4 recurrent chordoma (table S1). In addition, we also got 9 expressed long non-coding RNAs with the criteria of adjusted *p* values 0.05 and | log change | ≥ 2 (table S2). The number of underlying target mRNAs from miR-186-5p, miR-30c-5p, miR-151b, miR-125b-5p, and miR-1260a was 2346, 2377, 95, 1779, and 584 respectively (table S3, S4, S5, S6, and S7) by bioinformation analysis.

For verify the target mRNAs were the important factors that contributing to progression of chordoma, the target genes were intersected with the differential expressed mRNAs above. We found that AMOT, NPTX1, RYR3, and P2RX5 were the target protein mRNAs of miR-186-5p; NPTX1 was the target protein mRNAs of miR-125b-5p; and AMOT and TNFSF14 were the target protein mRNAs of miR-1260a (Fig. 5C, and Figure S4). The expression of AMOT, RYR3, and P2RX5 were lower in the miR-186-5p group than NC group while NPTX1 was contrast; The expression of NPTX1 were higher in the miR-186-5p group than NC group; The expression of AMOT and TNFSF14 were higher in the miR-186-5p group than NC group. And, the results were accordance with the mRNA array. The expression of AMOT, RYR3, P2RX5, and TNFSF14 were higher in recurrent chordomas than primary chordomas while NPTX1 was contrast. Then, we performed the luciferase reporter assay to confirm that miR- 186-5p could directly bind to the 3′-UTR of AMOT and miR- 125b-5p to NPTX1 in U-CH1 chordoma cells. And, the results showed that overexpression of miR-186- 5p and miR- 125b-5p significantly reduced luciferase activity of the reporter gene in wild type, but not mutant, indicating that miR-186- 5p directly targeted the AMOT 3′-UTR and miR-125b- 5p directly targeted the NPTX1 3′-UTR (Fig. 5D, and 5E).

### miR-30c-5p inhibits proliferation, migration, and invasion of chordoma

For further identify the effect of miR-30c-5p on chordoma cells, chordoma cells were transfected with miR-30c-5p mimics. RT-PCR result revealed that the experiment group has significantly high expression level than the contrast group (Figure S5A) in U-CH1 and UM-chor1 cells. Then, CCK-8 assay and EDU assay showed that the proliferation of U-CH1 and UM-chor1 cells was significantly inhibited compared with NC group (Figure S5B, S5C, S6A and S6B). In addition, wound healing assay and transwell experiment indicated that the migration and invasion ability of chordoma could attenuated by miR-30c-5p (Figure S5D, S5E, S5F, S6C, S6D, S6E and S6F). These results collectively suggested that miR-30c-5p inhibits cell proliferation, migration, and invasion.

### miR-151b inhibits proliferation, and invasion of chordoma

For further identify the effect of miR-151b on chordoma cells, chordoma cells were transfected with miR-151b mimics. RT-PCR result revealed that the experiment group has significantly high expression level than the contrast group (Figure S7A) in U-CH1 and UM-chor1 cells. Then, CCK-8 assay and EDU assay showed that the proliferation of U-CH1 and UM-chor1 cells was significantly inhibited compared with NC group (Figure S7B, S7C, S8A and S8B). In addition, wound healing assay and transwell experiment indicated that the migration and invasion ability of chordoma could attenuated by miR-151b (Figure S7D, S7E, S7F, S8C, S8D, S8E and S8F). These results collectively suggested that miR-151b inhibits cell proliferation, migration, and invasion.

## Discussion

Two groups were identified from the miRNA array by the machine learning method, which proven that chordoma has the distinct expression in the underlying progression mechanisms although most chordomas has a similar pathology[9]. This method gave us a new insight to the core of the progression mechanisms of chordoma. With its help, thirteen miRNAs were identified. And, after the transfection of corresponding miRNA mimics, we confirmed that miR-186-5p, miR-30c-5p, miR-151b, miR-125b-5p, and miR-1260a may be the important regulatory miRNAs in the development and progression of chordoma.

Contrast with our result, previous study has proven that miR-186-5p, miR-30c-5p, miR-151b, and miR-125b-5p play an anticancer role while miR-1260a play a cancer role in the development of cancers[6, 19–22]. In breast cancer, miR-186-5p was corrected with tumour size and tumour staging by downregulating CXCL13 [19]. As for the miR-30c-5p, miR-30c-5p inhibitors restored Bcl2 expression and inhibited apoptosis and increased colony and migration characteristic of glioma[6]. RUNDC3A-AS1 enhanced cell proliferation and inhibited cell apoptosis by targeting miR-151b to regulate the SNRPB expression[23]. miR-125b-5p inhibited cell proliferation, migration, invasion, and induced cell apoptosis in lung adenocarcinoma [21]. In another study, miR-1260a was downregulated when Glutaminase isoenzymes GLS was knocked down while miR-1260a was upregulated when GAB (Glutaminase isoenzymes GLS2 isoforms) was overexpressed [22].

To explore the mechanism by which the five miRNAs affect chordoma cells, bioinformatics tools and the mRNA expression array were used. With the help of multiMiR R package, the number of target mRNAs from miR-186-5p, miR-30c-5p, miR-151b, miR-125b-5p, and miR-1260a was 2346, 2377, 95, 1779, and 584 respectively. Furthermore, the underlying target mRNAs were confirmed by the mRNA array result and qRT-PCR experiment. Our previous report revealed that recurrent chordomas have a poor progression free survival than the primary chordomas (Fig. 5A) [24]. Herein, we did a mRNA sequence between primary and recurrent chordomas to select the underlying target mRNAs. After the intersection of the differential mRNAs and the target mRNAs above and the qRT-PCR experiments, we found that AMOT, NPTX1, RYR3, and P2RX5 were the target protein mRNAs of miR-186-5p; NPTX1 was the target protein mRNAs of miR-125b-5p; and AMOT and TNFSF14 were the target protein mRNAs of miR-1260a.

In the previous study, miR-497 could regulate the progression, migration, and invasion of osteosarcoma by targeting AMOT [25]. And, AMOT presents cancer role in the progression of osteosarcoma[26]. In another study, NPTX1 could inhibit cell proliferation through down-regulating cyclin A2 and CDK2 expression in colon cancer[27]. And, TNFSF14 could cause significant changes in vascular normalization and generation of tertiary lymphoid structures to regulate tumor environment[28]. LIGHT/TNFSF14 could promote osteolytic bone metastases in non-small cell lung cancer patients[29]. As for ryanodine receptor 3 gene (RYR3), miR-367 could regulate the expression of a reporter gene driven by the RYR3 3’UTR and the regulation was affected by the RYR3 genotype[30]. P2RX5 was seemingly associated with hereditary papillary histology[31]. In the future, the relationship between miRNAs and the target mRNAs still needs more efforts to validate.

In conclusion, miR-186-5p, miR-30c-5p, miR-151b, and miR-125b-5p could inhibits proliferation, migration, and invasion of chordoma while miR-1260a enhances proliferation, migration, and invasion of chordoma. Additionally, AMOT, NPTX1, RYR3, and P2RX5 were identified the target protein mRNAs of miR-186-5p; NPTX1 was identified the target protein mRNAs of miR-125b-5p; and AMOT and TNFSF14 were identified the target protein mRNAs of miR-1260a. However, the identical correction between them was unclear. More efforts need be done to reveal it in the future.

### Electronic supplementary material

Below is the link to the electronic supplementary material.

Supplementary Material 1

Supplementary Material 2

## Data Availability

Raw sequencing data was deposited in National Genomics Data Center (NGDC, http://bigd.big.ac.cn/) and the BioProject number was HRA004915 (NGDC - GSA for Human (cncb.ac.cn)). Any additional information is available from the correspondence author.
