# Supplementary material for: Function and regulation of miR-186-5p, miR-125b-5p and miR-1260a in chordoma
Source: BMC Cancer. 2023 Nov 27;23:1152. doi: 10.1186/s12885-023-11238-x (PMC10680222; doi:10.1186/s12885-023-11238-x)
Supplement: Supplementary file 2 — Supplementary Material 2 [file 12885_2023_11238_MOESM2_ESM.docx]

**Title:** Function and Regulation of miR-186-5p, miR-125b-5p and miR-1260a in Chordoma

**Authors:** Xulei Huo^1^, Ke Wang^#1^, Bohan Yao^1^, Lairong Song^1^, Zirun Li^2^, Wenyan He^2^, Yiming Li^3^, Junpeng Ma^1^, Liang Wang^1^ and Zhen Wu^#1^

**Affiliation:** ^1^Department of Neurosurgery, Beijing Tiantan Hospital, Capital Medical University, Beijing, China. ^2^ China National Clinical Research Center for Neurological Diseases, Beijing, China. ^3^Department of Neurosurgery, Tianjin Medical University General Hospital.

^#^The corresponding authors

**Running title:** A analysis of miRNA in chordoma

**Keywords**

chordoma; miRNA; migration; proliferation; mRNA

**Financial support:** This work was supported by National Natural Science Foundation of China (Nos. 62027813, 2022YFE0112500, and 8180100922), the Beijing Municipal Science and Technology Commission (No. 7192056), and the Natural Science Foundation of Beijing (No. J180005).

**Corresponding authors:** Zhen Wu, email: [wuzhen1966@aliyun.com](mailto:wuzhen1966@aliyun.com), Tel: +86-15601125551, Department of Neurosurgery, Beijing Tiantan Hospital, Capital Medical University, Nansihuanxilu 119, Fengtai District, Beijing, 100070, China; Ke Wang, wangke@bjtth.org.

**Conﬂict of Interest:** All authors pronounce that there are no any commercial or ﬁnancial relationships that could be interpreted as a potential conﬂict of interest.


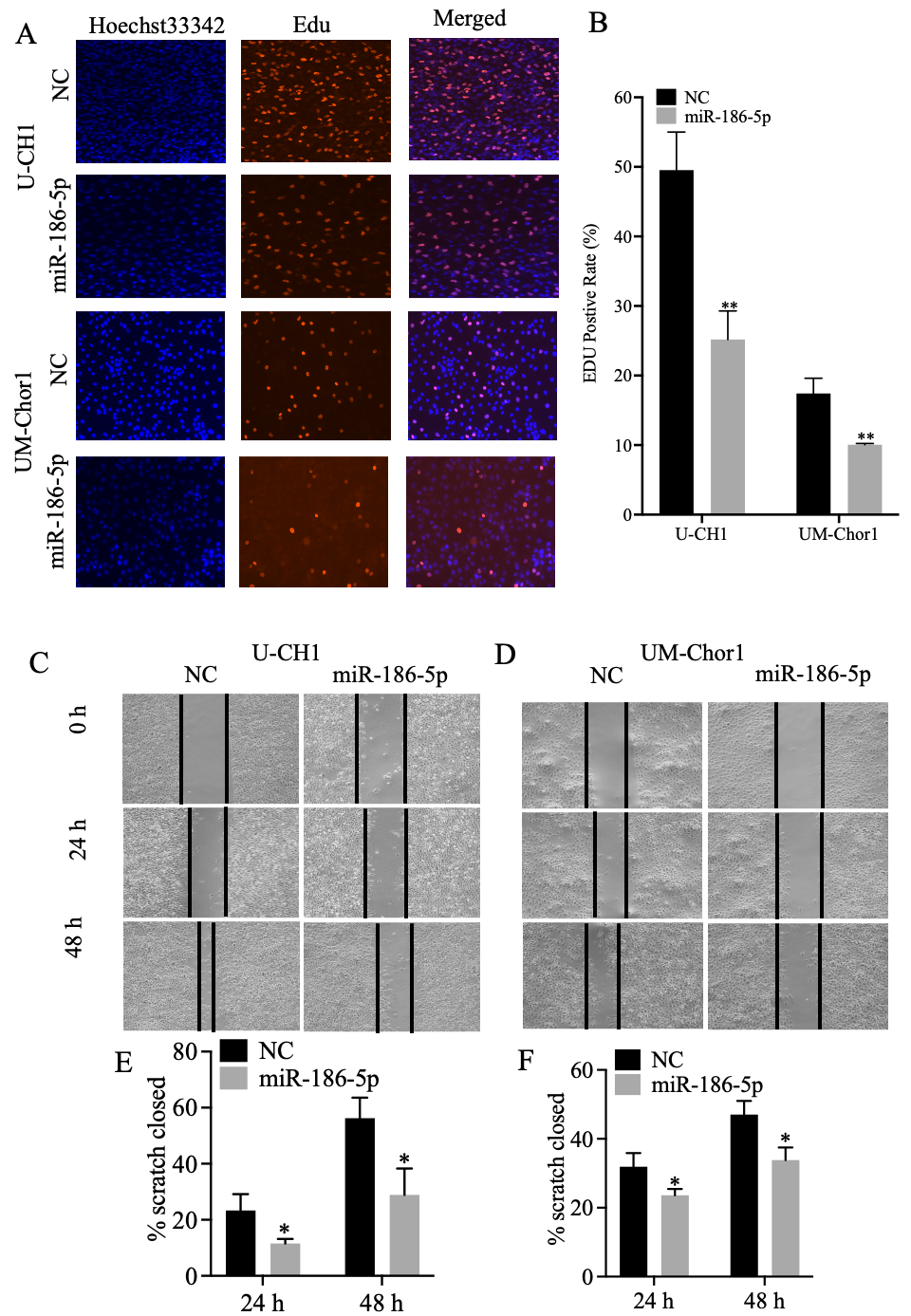


Figure S1. miR-186-5p inhibits the migration, invasion and proliferation of U-CH1 cells and UM-Chor1 cells. **A** Representative pictures and **B** quantitative data of EDU assay in U-CH1 cells and UM-Chor1 cells from the miR-186-5p group and the NC group. **C**, **D** Representative pictures and **E, F** quantitative data of wound healing assay in U-CH1 cells and UM-Chor1 cells from the miR-186-5p group and NC group. Each experiment was performed in in triplicate and repeated three times. Results are presented as mean ± standard deviation.


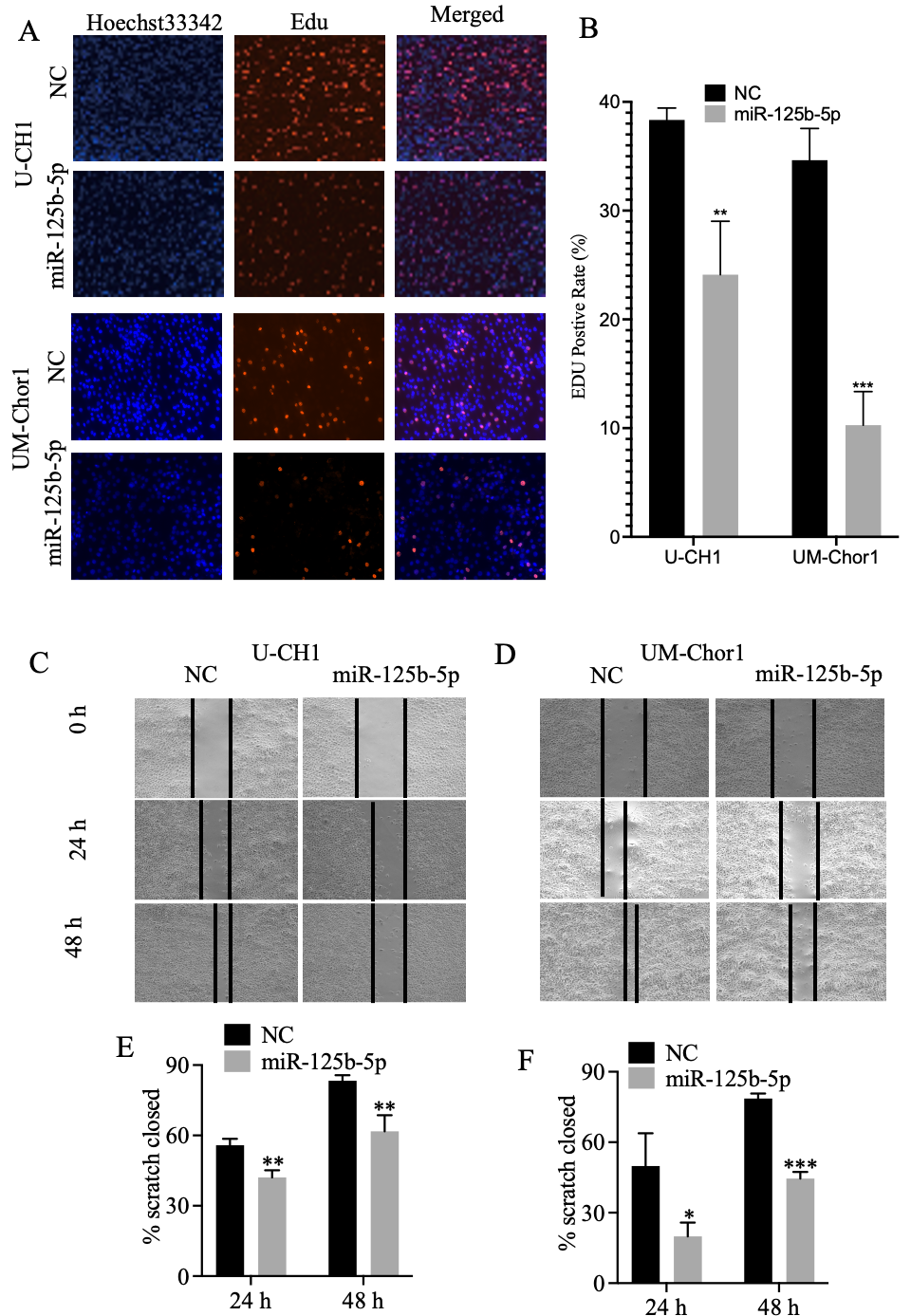


Figure S2. miR-125b-5p inhibits the migration, invasion and proliferation of U-CH1 cells and UM-Chor1 cells. **A** Representative pictures and **B** quantitative data of EDU assay in U-CH1 cells and UM-Chor1 cells from the miR-125b-5p group and the NC group.**C**, **D** Representative pictures and **E, F** quantitative data of wound healing assay in U-CH1 cells and UM-Chor1 cells from the miR-125b-5p group and NC group. Each experiment was performed in in triplicate and repeated three times. Results are presented as mean ± standard deviation.


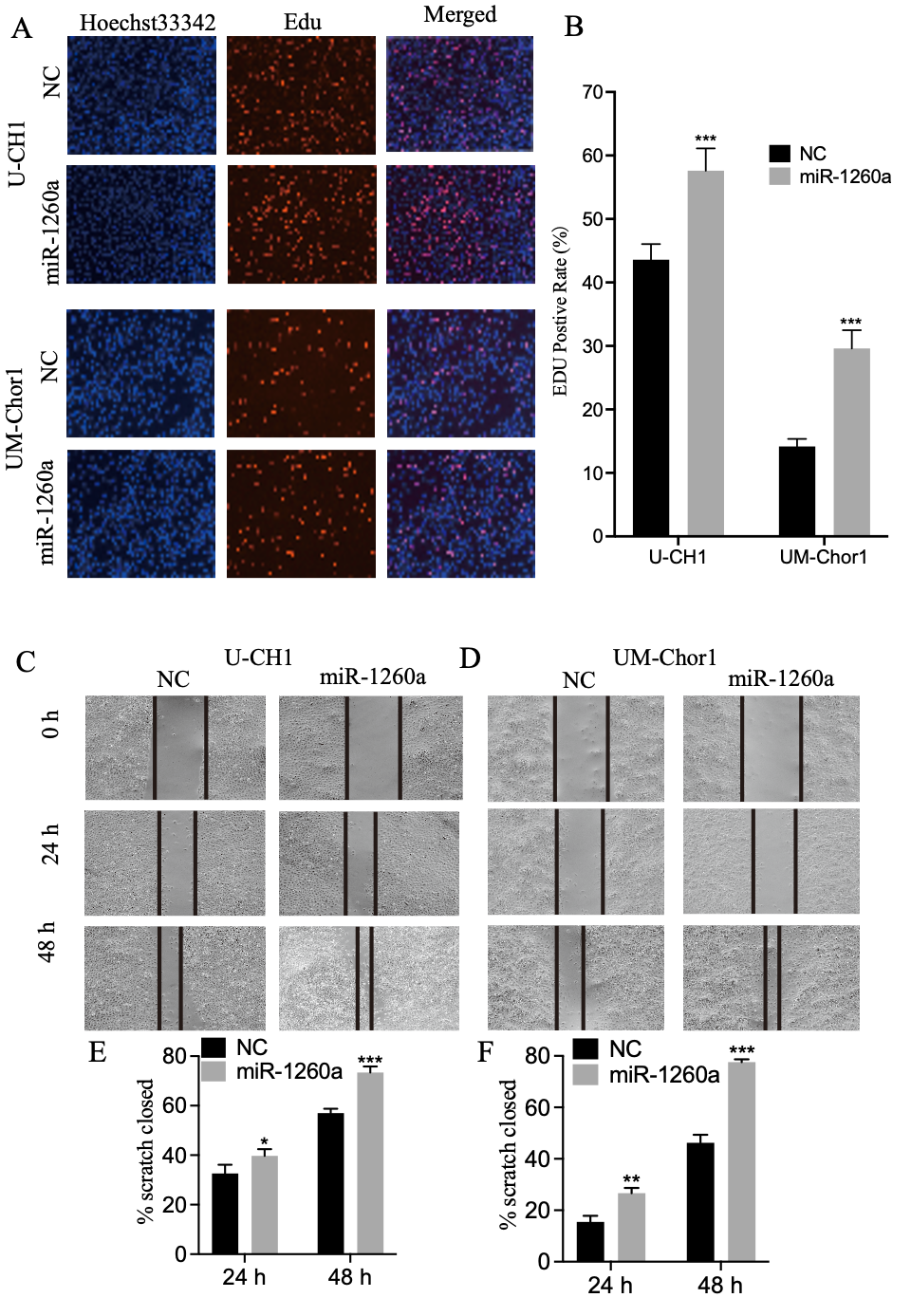


Figure S3. miR-1260a enhances the migration, invasion and proliferation of U-CH1 cells and UM-Chor1 cells. **A** Representative pictures and **B** quantitative data of EDU assay in U-CH1 cells and UM-Chor1 cells from the miR-1260a group and the NC group. **C**, **D** Representative pictures and **E, F** quantitative data of wound healing assay in U-CH1 cells and UM-Chor1 cells from the miR-1260a group and NC group.

Each experiment was performed in in triplicate and repeated three times. Results are presented as mean ± standard deviation.


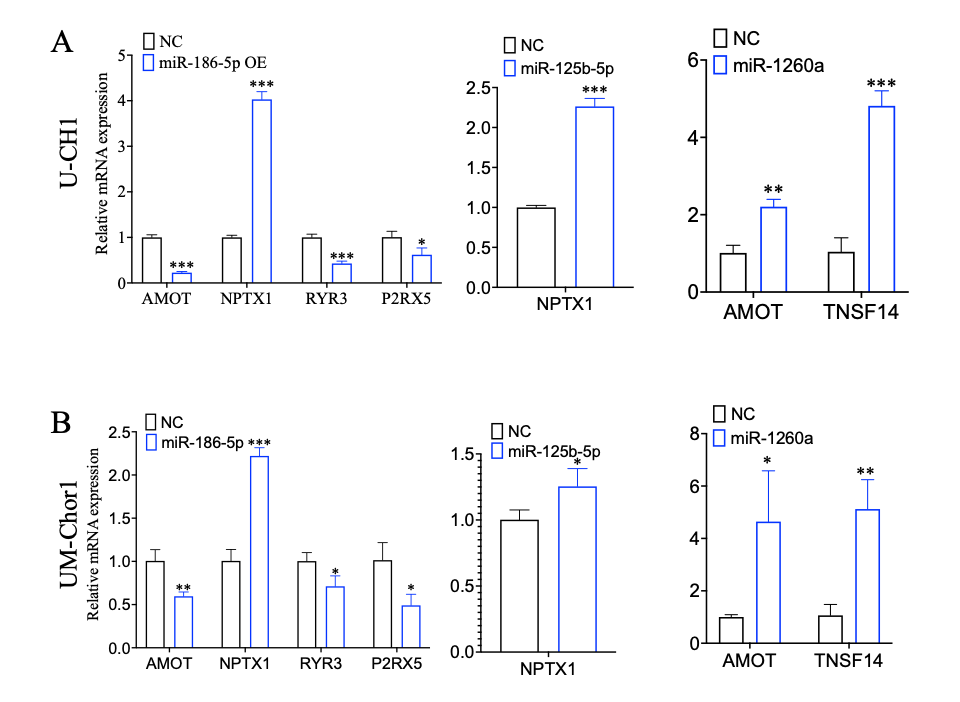


Figure S4. qRT-PCR results of the targets of miRNAs. **A, B** revealed that AMOT, NPTX1, RYR3, and P2RX5 were the target protein mRNAs of miR-186-5p; NTPTX1 was the target protein mRNAs of miR-125b-5p; AMOT and TNFSF14 were the target protein mRNAs of miR-1260a. GADPH was served as the internal control. Each experiment was performed in in triplicate and repeated three times. Results are presented as mean ± standard deviation.


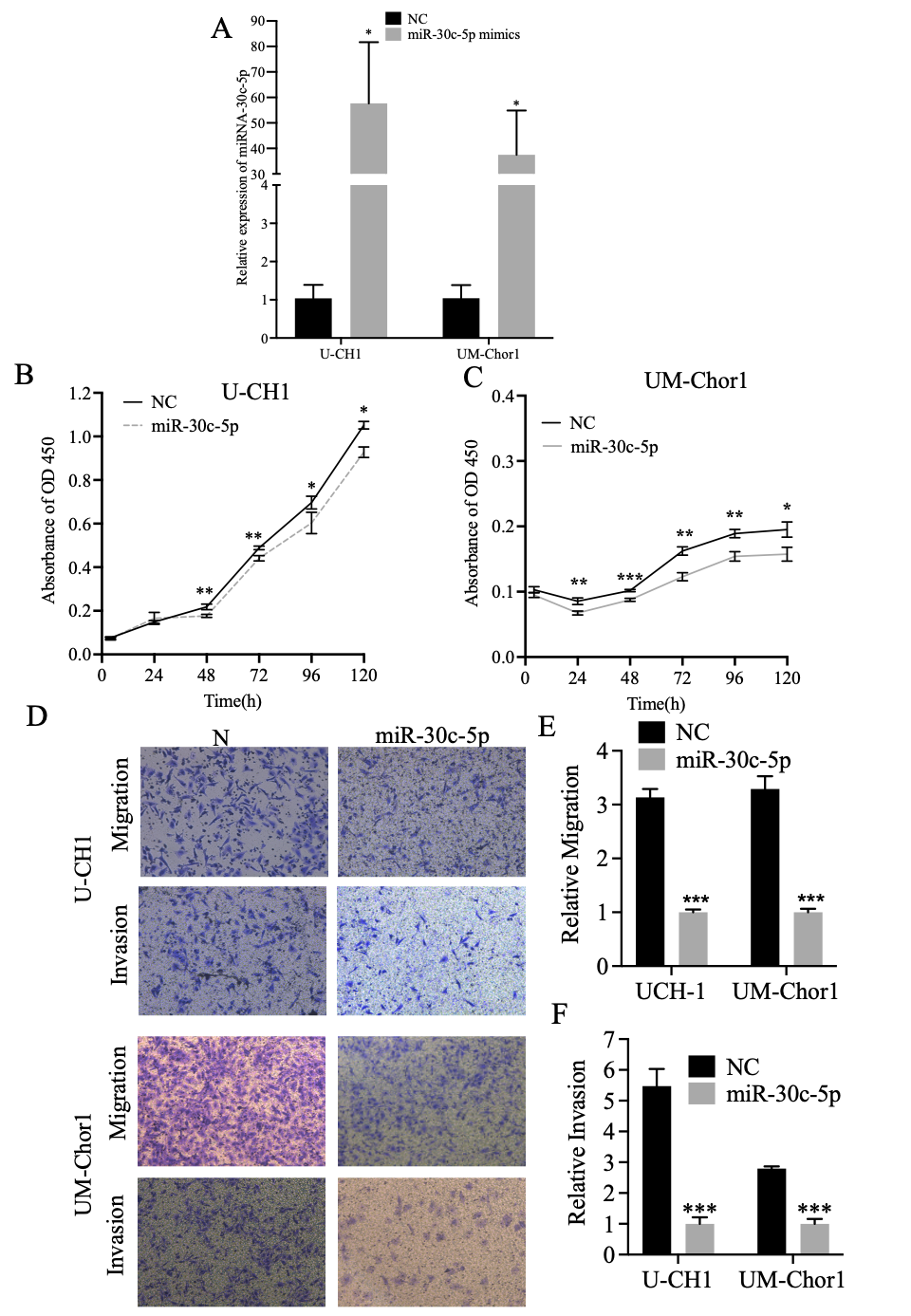


Figure S5. miR-30c-5p inhibits the migration, invasion and proliferation of U-CH1 cells and UM-Chor1 cells. **A** qRT - PCR results of miR-30c-5p in U-CH1 cells and UM-Chor1 cells from the miR-30c-5p overexpression (miR-30c-5p) group and NC group. U6 was served as the internal control. **B** Representative pictures of real-time proliferation assay in U-CH1 cells and **C** UM-Chor1 cells from the miR-30c-5p group and the NC group. **D** Representative pictures and **E, F** quantitative data of transwell assay in U-CH1 cells and UM-Chor1 cells from the miR-30c-5p group and NC group. Each experiment was performed in in triplicate and repeated three times. Results are presented as mean ± standard deviation.


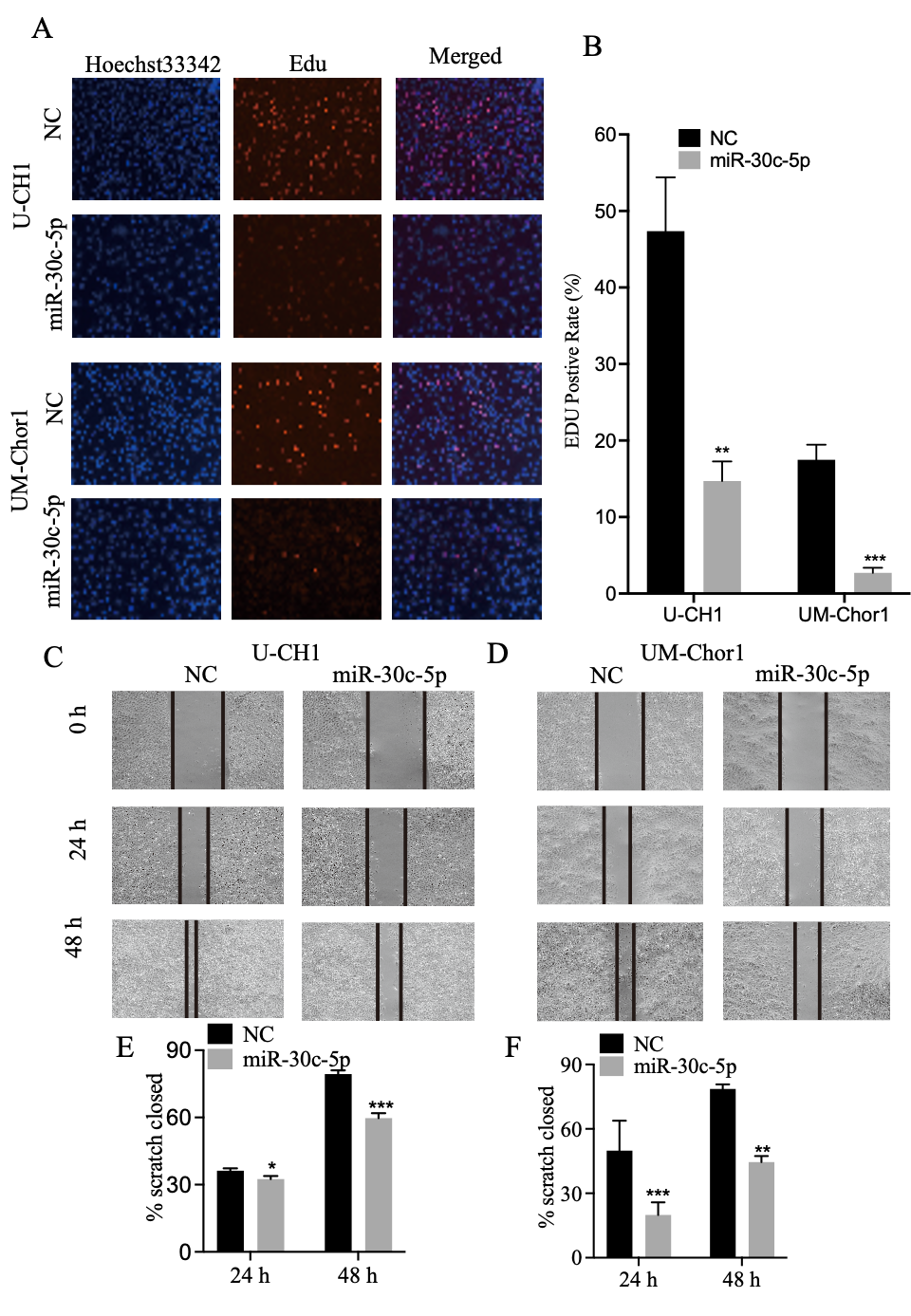


Figure S6. miR-30c-5p inhibits the migration, invasion and proliferation of U-CH1 cells and UM-Chor1 cells. **A** Representative pictures and **B** quantitative data of EDU assay in U-CH1 cells and UM-Chor1 cells from the miR-30c-5p group and the NC group. **C**, **D** Representative pictures and **E, F** quantitative data of wound healing assay in U-CH1 cells and UM-Chor1 cells from the miR-30c-5p group and NC group. Each experiment was performed in in triplicate and repeated three times. Results are presented as mean ± standard deviation.


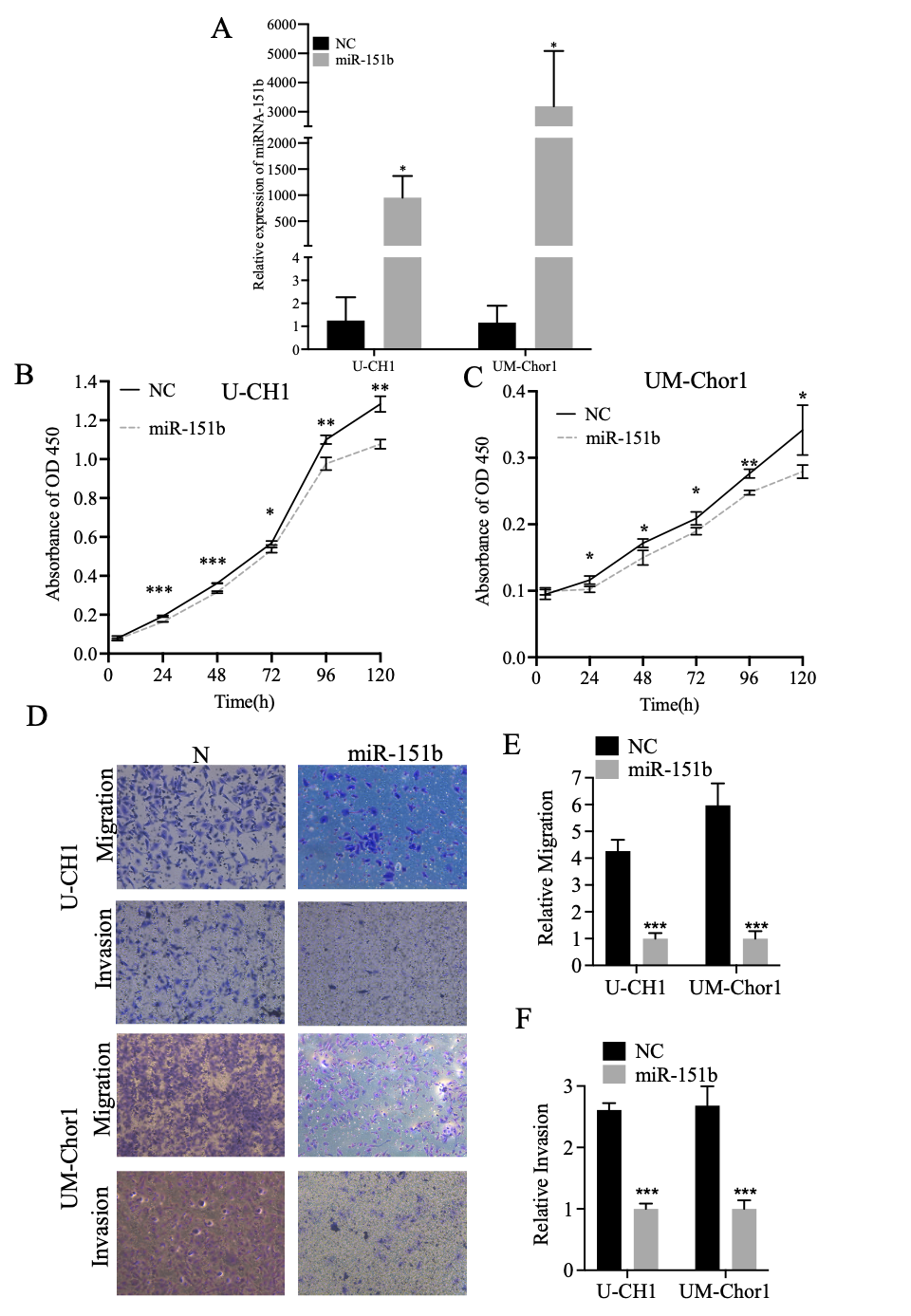


Figure S7. miR-151b inhibits the migration, invasion and proliferation of U-CH1 cells and UM-Chor1 cells. **A** qRT - PCR results of miR-151b in U-CH1 cells and UM-Chor1 cells from the miR-151b overexpression (miR-151b) group and NC group. U6 was served as the internal control. **B** Representative pictures of real-time proliferation assay in U-CH1 cells and **C** UM-Chor1 cells from the miR-151b group and the NC group. **D** Representative pictures and **E, F** quantitative data of transwell assay in U-CH1 cells and UM-Chor1 cells from the miR-151b group and NC group. Each experiment was performed in in triplicate and repeated three times. Results are presented as mean ± standard deviation.


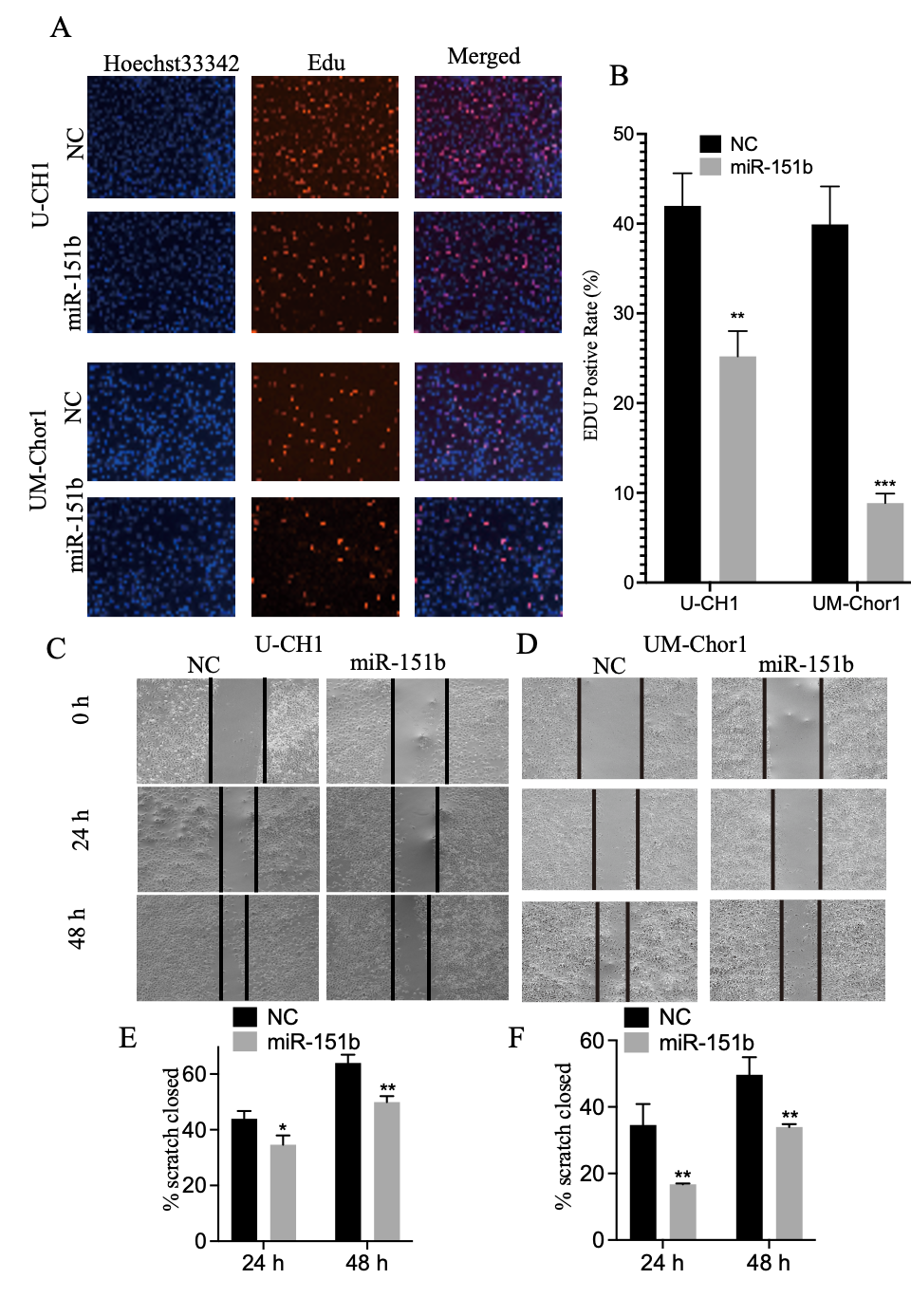


Figure S8. miR-151b inhibits the migration, invasion and proliferation of U-CH1 cells and UM-Chor1 cells. **A** Representative pictures and **B** quantitative data of EDU assay in U-CH1 cells and UM-Chor1 cells from the miR-151b group and the NC group. **C**, **D** Representative pictures and **E, F** quantitative data of wound healing assay in U-CH1 cells and UM-Chor1 cells from the miR-151b group and NC group. Each experiment was performed in in triplicate and repeated three times. Results are presented as mean ± standard deviation.
